# Reproducing experiential meaning in translation: A systemic functional linguistics analysis on translating ancient Chinese poetry and prose in political texts

**DOI:** 10.3389/fpsyg.2022.1029187

**Published:** 2022-11-24

**Authors:** Qiannan Liu, Xiaolong Li

**Affiliations:** ^1^School of Languages, Nanjing Normal University of Special Education, Nanjing, China; ^2^College of International Languages and Cultures, Hohai University, Nanjing, China

**Keywords:** systemic functional linguistics, transitivity, translation shift, experiential meaning, Chinese-English translation

## Abstract

Ancient Chinese poetry and prose (ACPP) are the essence of traditional Chinese culture and also literary gems of the world. Despite the long tradition of previous Chinese Presidents using ACPP in their political addresses, the translation work on the three volumes of *Xi Jinping: The Governance of China* with much ACPP still poses a daunting challenge to the translation of literary texts in non-literary texts. As experiential meaning is inherent in all kinds of texts, literature, and non-literature, this study, drawing on the sights of Halliday's experiential mode of meaning and register theory in Systemic Functional Linguistics (SFL), explored how the ACPP's experiential meaning was rendered in the political texts, and factors motivating transitivity patterns and shift tendencies. Statistics showed that the translations tend to reproduce the experiential meaning such that it involves more representations of the flow of events and the creation of relationships among entities, focusing more on construing the experience of the physical world rather than personal emotions, feelings, or cognition. Besides, the target text is also inclined to simplify the experiential meaning by compressing and omitting unnecessary transitivity processes and the participant and circumstance nominalization. This research found that the factors responsible for such tendencies include the features of political texts, language differences, the distribution of process types, the delicacy of the transitivity system, and register variations of fields of activity. With these findings, the study is expected to contribute to the SFL approach of studying literary translation in non-literary texts through the prism of experiential meaning and offer guidance in the translation of political literature with ACPP.

## Introduction

Ancient Chinese poetry and prose (ACPP) embody the profound and ancient culture and wisdom of the Chinese nation, representing the knowledge and rational thoughts developed over several millennia. Quoting ACPP in their political addresses has been a long tradition for Chinese presidents. The same is true for the current paramount leader of China, Xi (2014a, 2017a, 2020a), with his recent works *The Governance of China* (hereinafter referred to as *Governance*) Volumes I to III, an authoritative source of Xi's political philosophies and collections of China's policies in the last decade. As China continuously advances major-country diplomacy with Chinese characteristics and moves closer to the center of the world stage, the *Governance* volumes, with translations of 33 languages and distribution in more than 170 countries and regions (Huaxia, 2021), have become a critical window for the world to learn about China over the past decade. When it comes to cultural outreach, one of the prominent features of Xi's book is the frequent quotation of ACPP. These citations, from the Hundred Schools of Thought to the Confucian classics, help interpret major concepts and critical ideas proposed by President Xi, incorporating impressions on the original readers, resonanating with many. However, concerning the translation of much ACPP in *Governance*, how to render literary texts in political texts is still a challenge, in the absence of much research.

The study of reproducing ACPP in *Governance* has substantial empirical and practical value. The volumes comprise political speeches and writings that shed light on Xi Jinping's Thoughts on Socialism with Chinese Characteristics for a New Era, his philosophical interpretation of many political issues regarding China's development paths, and theories and institutions of China's economy, politics, diplomacy, culture, rule of law, the Party's leadership, etc. The ACPP quoted in Xi's works are literary texts, including the *Shijing, Chuci*, the poetry from the Han Dynasty to the Qing Dynasty, Early Chinese prose written in the Spring and Autumn Period and the Warring States Period, Han prose, *Guwen* in the Tang and Song dynasties and other forms of prose created before the modern Chinese times. The major difference between literary and non-literary texts “is that the first comprises the world of the mind and the imagination; the second, the world of reality, of facts and events” (Newmark, 2004, p. 10). Such a difference makes us wonder how to translate the meaning behind “the world of mind and the imagination” when they are parts of the texts comprising the meaning behind “the world of reality”. For this, we can explore the reproduction of the experiential meaning of ACPP in political texts. Experiential meaning embodies the author's or speaker's understanding of the experience of the world, according to Halliday's Systemic Functional Linguistics (SFL) (Halliday, 1994; Halliday and Matthiessen, 2004, 2014). This mode of meaning, as SFL theorists believed, carries basic information, and serves as the foundation for all kinds of texts to form their meanings, or more specifically, the metafunctional meanings inherent in language itself. Experiential meaning is realized through the lexicogrammatical system of transitivity, a network proposed by Halliday to investigate how language construes the world of experience at the micro linguistic level, i.e., the experiential meaning is the innate meaning of all text types, literature, and non-literature, as all of them comprise the author's meaning-making of the experience in the world. Therefore, despite the principal difference between literary and non-literary texts, SFL's experiential mode of meaning with its linguistic analytical approach to the transitivity system enables us to study how the ACPP was rendered in Xi's representative works. Such an approach is thus of empirical significance for this research, to reveal the applicability of SFL in studying the translation of literary texts in non-literary texts, and offer practical guidance to translators rendering literary citations in political texts.

To do so, through a comparison between the Source Text (ST) and Target Text (TT) in Xi's *Governance* volumes, this research explores the following issues:

1) What are the transitivity patterns of the translation when it reproduces the experiential meaning of the source text?
  a. Are there any transitivity shifts in the translations?
  b. To what extent was the experiential meaning shifted in the translations?
2) What types of transitivity shifts tend to be involved in the translations?
3) What are the possible factors that motivate the transitivity shift tendencies?

To answer these questions, drawing on the insights of Halliday's SFL, this study, through a case study of *Governance* Volumes I to III, describes the transitivity patterns and major transitivity shift tendencies of reproducing the experiential meaning of ACPP in political texts and discusses potential factors motiving such tendencies.

## Literature review

SFL has been recognized as an important research resource in translation studies since the 1950s when Halliday's SFL theory was still in its infancy, and even the creation of Systemic Functional Translation Studies (SFTS) for the applied and theoretical research influenced by SFL. To illustrate the foundations relevant to our research, and to locate the limitations and gaps in previous works to justify the current study, this section provides an overview of a) how experiential meaning, transitivity, and register are located and connected with the context in SFL, b) problems and room for further improvement in studies on the application of transitivity in translation, and c) previous studies on the translation of ACPP cited in *Governance* volumes.

### Locating experiential meaning, transitivity, and registering in SFL

Experiential meaning refers to one of the major metafunctional modes of meaning that construe “the model of experience” (Halliday and Matthiessen, 2004, p. 61). Considering language as a meaning-making system in the sense of SFL, there are four modes of metafunctional meanings inherent in language, which are fulfilled along with the realization of three metafunctions, viz., ideational metafunction, realized in expressing experiential and logical meanings, interpersonal metafunction (enacting relationships in a sociocultural context through interpersonal meaning), and textual metafunction (facilitating the first two functions coherently in textual meaning). They are termed as such because they are innate in language and are indispensable factors that can themselves be used to analyze language. Among them, experiential meaning embodies the original writer's understanding of a certain experience of the world, i.e., experiential meaning is the innate meaning for all kinds of texts, be it literature or non-literature, as they all comprise the author's meaning-making of the world. Therefore, experiential meaning can facilitate analysis of the translation of ACPP in political texts, regardless of the differences in text genres. Further, experiential meaning is the primary parameter in the metafunction system as other meanings cannot be registered without the representation of the experience of the world in the first place (e.g., Matthiessen, 2014, p. 277; Huang, 2016, p. 300–301).

Transitivity is the lexicogrammatical network and has been proposed by Halliday and Matthiessen (2004, 2014) as the direct resource for the realization of experiential meaning in terms of the rank of a clause within the ideational metafunction. In other words, we can see how experiential meaning is represented by analyzing the linguistic forms through which the meaning is realized, i.e., the transitivity system. Such a lexicogrammatical system is greatly influenced by other essential strata in SFL, i.e., the context that links lexicogrammar with semantics. To conceptualize the context more pragmatically, the register theory was developed by Halliday and Matthiessen (2004, 2014), Wang et al. (2019). Matthiessen (2015) defined register as “a functional variety of language, i.e., the meanings at risk in a given type of context” and offered specific parameters to examine contextual elements in texts, thus clearly enunciating the theoretical framework for analyzing the representation of experiential meaning lexicogrammatically and contextually in SFL.

**Figure 1:**
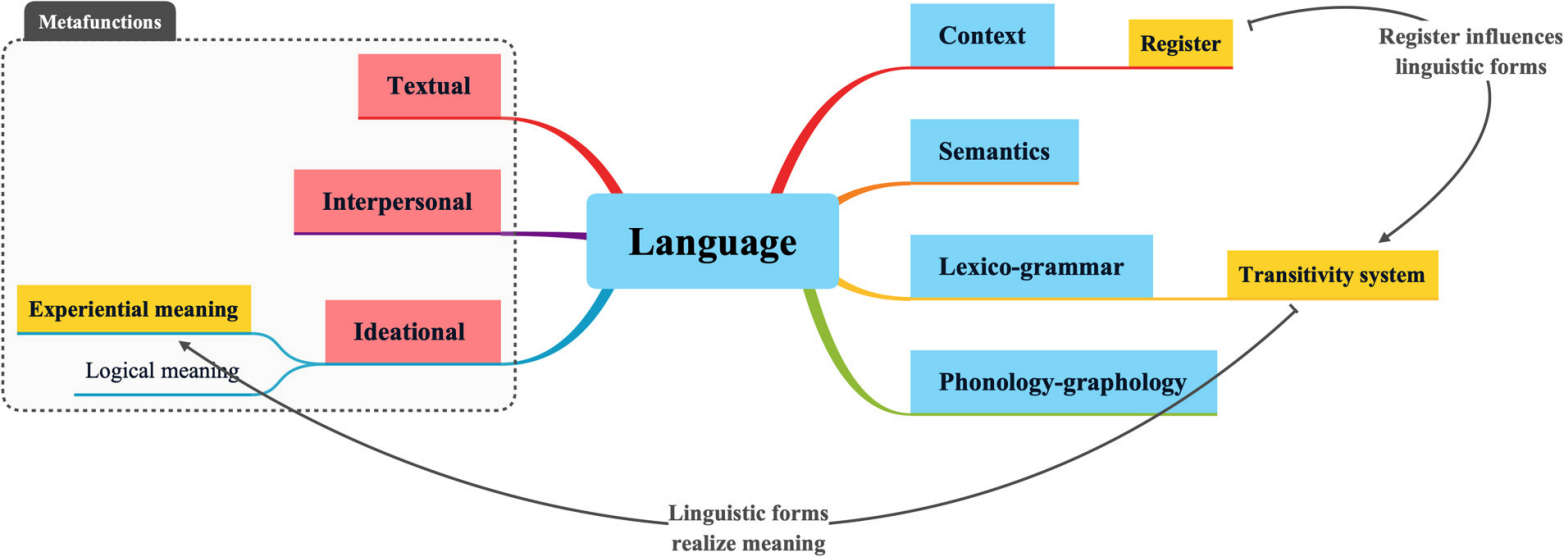
Language architecture in SFL.

Overall, in the sense of SFL, language is conceived as a meaning-making system that can realize its experiential meanings through the choices of transitivity linguistic forms, which are then influenced by the contextual variables of the register (see Figure 1). Such basic ideas later form the foundations for SFTS as an applicable theory, as SFL shows a “strong interrelation between the linguistic choices, the aim of the form of communication and the sociocultural framework” (Munday et al., 2022, p. 122).

### The application of transitivity in translation

#### Transitivity applied in metafunctional translation equivalence and shift

Transitivity systems have been widely used in comparative translation studies for the metafunctional equivalence and shift, laying foundations for further studies with various possible changes of and among metafunctional meanings and factors to such shifts. Thanks to Catford's (1965) research, the earliest monograph on translation with SFL, translation equivalence and translation shift were defined regarding rank and level of SFL's organization of language, based on Halliday's (1961) early version of Systemic Functional Grammar (SFG). Since then, translation equivalence and shift have become important paradigms in SFTS. Hatim and Mason (1997, p. 6–9) through the comparative analysis of transitivity patterns of a fiction and its translation, suggested the shift of transitivity may lead to the change of metafunction. Inspired by their study, Munday (2002, p. 79) by employing a flexible method of SFL to investigate metafunctional meanings, a corpus linguistic approach to exam the whole texts, and ‘locating the results within the wider publishing, political and sociocultural contexts', proposed a systematic and replicable model for analyzing the ST and TT. Based on the model, he applied transitivity to explored the shifts between passive and active voices and points out possible motivations, focusing on the ideology of the translator and the publisher. Such analyses can be ‘extremely useful for the translator in identifying important elements in an ST and seeing how they create meaning in a specific cultural and communicative context' (Munday et al., 2022, p. 124). Matthiessen (2001) was particularly interested in metafunctional equivalence and shift and summarized five types of metafunctional variations (including both equivalence and shift) across and within ranks, showing possible transitivity shifts at the level of rank. Later, Matthiessen (2014) examined the metafunctional shift more closely and proposed the matrix of metafunctional translation shifts to illustrate the two types of shifts: those within a metafunction and those moving from one metafunction to the other. However, this matrix still needs to be completed and proven. What the matrix indicates are the tendencies of metafunctional shifts shown in empirical analysis, while in principle, all possible shifts can occur (Wang et al., 2019). Therefore, Matthiessen's observation of the patterns of metafunctional translation shifts, including experiential metafunction translation shifts, a model of trial and error based on the analysis of multiple translation cases can be very inspiring, enabling future studies in this field.

Domestically, there have been an increasing number of scholars applying SFL in translation studies, with certain empirical research dedicated to translation from the perspective of metafunctional equivalence or shift. Among those, few pay attention to the translation of experiential meaning, causing “imbalanced metafunctional considerations” (Wang and Ma, 2021, p. 77–78). Nevertheless, the prior works can be instructive for the current study in terms of the possible types of transitivity shifts and motivations to transitivity shifts. For instance, Shang (2005, p. 113–115) studied E–C translation shifts in political texts by analyzing the metafunctional shifts of four political addresses by previous U.S. and UK presidents. She found three tendencies of ideational meaning shifts: adding and extending participants and process types, omitting and combing them, and modifying and substituting the two components. This research may be limited as it analyzed only the nuclear transitivity (i.e., process and participant components), thus ignoring the circumstance shifts which also play a part in changing meanings. Hence, the current research included the analysis of all three transitivity components for greater effectiveness. However, what can be inspired is that Shang (2005, p. 15) not only explored “the local variables at text or language system level that might cause or motivate shifts of the specific linguistic forms or expressions between the parallel texts”, but also investigated how the three register variables can affect translation shifts, thus putting contextual factors into consideration. Similarly, unlike the most empirical metafunctional translation studies that only probe into translators' lexicogrammtical choices, Ma and Wang (2020) identified three process type shifts with transitivity systems, and linked such an analysis with graphological, phonological and contextual ones and other lexicogrammtical analyses, proposing a comprehensive and applicable framework to exam the choices poetry translators would face.

#### Transitivity applied in literary translation

When reviewing previous studies on the application of transitivity in literary translation studies, especially the translation of ACPP, we observe a salient dispute about the extent to which the experiential meaning can be rendered through the realization of the formal equivalence of transitivity systems between Chinese and English. Some (e.g., Huang, 2002, 2003, 2004; Zhang, 2010) suggest that the formal equivalence of experiential meaning should be fulfilled to offer a better literary translation. Further, the equivalence of experiential metafunctional meaning is likely to be registered if the ST's transitivity pattern can be constant, to the extent of the TT. Some others (e.g., Zhang, 1999; Zhao, 2006; Zhao J., 2017; Wang and Ma, 2021) believe the formal equivalence of the transitivity system is not simply equal to the equivalence of experiential meaning. Instead, there is a complex relationship between the linguistic choice of the transitivity process type and reproducing experiential meaning in the TT. This is because the configuration of the transitivity process tends to change in the TT, as the reconstructing of experiential meaning is constrained by such factors as the language conventions in the target language society, the regenerating of other metafunctional meaning, and the contextual elements including the field, tenor, and mode which have interactions with the three metafunctions. Thus, it should be theoretically significant to explore the translation of the experiential meaning of the ACPP in Xi's volumes from the perspective of transitivity translation equivalence and shift.

A drawback of previous studies on literary translation from the SFL perspective is that while much research (e.g., Chen, 2009; Wang, 2014; Zou, 2015; Wang and Ma, 2020) focuses on the renderings of novels and ancient Chinse poetry, only a few authors consider the translation of certain Chinese classic prose, regardless of the topic, on the reconstruing experiential meaning of ACPP. Several such studies focus on the early Chinese prose of the Spring and Autumn and the Warring States Periods, like *The Analects of Confucius, Mencius*, and *Zhuangzi*, whilst the other classical prose developed during Chinese history is barely studied, including the *Pianwen*, Han prose, and *Guwen*, leaving potential space for the current study to fill.

Overall, one benefit of reviewing the aforesaid studies is that first, while research often suggests an ideal situation where metafunctional equivalence can be pursued through the fulfillment of formal equivalence based on SFL's principle of “form realizing meaning”, there can be reasonable shifts caused by other inter-linguistic and extra-linguistic elements in the wider environment of translation. What is also instructive from the previous studies is that contextual elements should be taken into consideration, as literary translation in political texts is not the same as pure literary translation. Further, the paucity of studies on the ACPP translation from the angle of reproducing its experiential meaning leaves room for exploration.

### Translation of ACPP in Xi's volumes of *Governance*

Two problems were noticed in our review of previous studies on the translation of Xi's works of *Governance*. First, though the past few years have witnessed an increasing number of studies involving the translation of Xi's books, few are dedicated to the Chinse–English translation of ACPP quoted in them. Much of the research explores the translation strategies and skills of the volumes or their specific linguistic features, including the translation of Chinese-specific expressions, or Chinese culture-loaded words, though involving the ACPP. Second, although various theories, the –additional translation theories or the modern ones–were adopted from different research perspectives, SFL was barely employed by them in translating Chinese-specific expressions or the ACPP in Xi's works, thus offering avenues for this study to explore.

Despite the paucity of studies directly linked to the subject of this study, inspiration can be gained from various studies which share common points to facilitate this study. Similar conclusions arrived at by different authors on the English translation strategies of allusions and expressions with Chinese characteristics, ACPP included, can be instructive for this study to examine the extent to which the experiential meaning may be changed. For instance, Zhu (2020), through a corpus-based textual analysis, summarized factors to the successful translations of allusions in *Governance* Volume I, including the poetry and prose, and calculated the frequency and proportion of translation approaches. Zhao X. (2017), Zhao (2018) integrated theories of cultural and sociological approaches and systematically studied the translation norms and strategies of five types of texts, comprising the Chinese-specific cultural items and ACPP in the central committee literature, including the first volume of *Governance*. They all drew similar conclusions regarding the tendency of translation strategies used for rendering the allusions of ACPP and unique Chinese expressions in *Governance* volumes: “a flexible use of translation methods” with literal translation or foreignization as the major methods, supplemented by other approaches, such as free translation, domestication, simplification, cultural alternatives, and offering extra explanation within the text (Zhu, 2020, p. 89). A literal translation is closely connected to a formal equivalence which, according to Nida (1964), seeks the closest match to the ST in both content and structural form, while free translation does not prioritize the maintenance of the original form. Such a conclusion can favor this study by offering a preliminary knowledge of how much the TT may be shifted regarding the experiential meaning.

## Theoretical framework

With regard to the theoretical framework, this research adopted a mixed method of the experiential mode of meaning, transitivity system, and register theory in SFL. This section will provide notions of English and Chinese transitivity grammars, types of transitivity shift for comparative analysis, and basic ideas of register theory to elaborate the framework.

### English and Chinese transitivity grammar

Regarding the notion of transitivity in SFL, unlike the transitivity in traditional grammar that seeks superficial structural features of texts in terms of the relationship between a verb and its object, Halliday's transitivity theory considers a clause as an analytical unit and can thus bring the semantic, contextual, and textual meanings into consideration. The English transitivity system developed by Halliday and Matthiessen (2014) has become one of the mainstream analytical frameworks in translation studies, with its strong power of explanation and the most comprehensive theoretical network employed so far in analyzing the transitivity of languages. This system was employed in this study too.

According to the two scholars (ibid.), experiential meaning is realized at the lexicogrammatical level through transitivity, where experiences are construed as the flow of events in clauses. A clause consists of three components:

a) Process.
b) Participants in the process.
c) Circumstances in the process.

Halliday (1994), Halliday and Matthiessen (2004, 2014) focused on the core element—**process—**often realized by the verb or verbal group, and categorized it into six English process types. Among them, there are the core process types used most commonly in English: the **material process** that construes what is happening or what is being done in the event; the **mental process** construing the experience of the world of consciousness; and the **relational process** used to characterize and identify the relationships among entities. The other less common ones are the subsidiary process types: the **behavioral process** which construes physiological and psychological behavioral experiences like breathing, coughing, and sleeping; the **verbal process** which creates a narrative, and the **existential process** which shows the existence of things. As for the detailed classification of English process types, the subtypes of the three major processes and the specific examples of verbs for the six processes are provided in Supplementary Table 1, according to Halliday and Matthiessen (2014, p. 169–248). **Participants** and **circumstances** as complementary elements in English clauses are usually realized by the nominal group (or noun) and the adverbial group, respectively. Each process type has a model for construing a particular domain of experience, with its typical configuration of participants and circumstances.

As for the Chinese transitivity system, despite their structural differences, Chinese and English share numerous commonalities in transitivity, particularly in processes, due to the similarity of human experiences, leading to similar sentence patterns and process types (Zhao, 2006). Processes barely change despite cultural differences, for it is the participant and circumstance components that mainly load the sociocultural elements. Several other core studies (e.g., Halliday and Matthiessen, 1999; Li, 2004a,b; Halliday and Webster, 2005; Peng, 2011) also proved that the transitivity systems of Chinese and English are proximate, despite superficial differences in the linguistic strata. Therefore, so far, the classification of the six Chinese process types remains the first choice for the most comparative Chinese–English translation studies (e.g., Huang, 2003; Si and Li, 2013; Wang and Ma, 2020), identical to Halliday's six English processes. Such a Chinese transitivity analytical approach can provide items comparable to the same classification English transitivity system, offering clearer results of how the translation is shifted from the original text. So, to make a comparative translation study between the two languages, we employed a six-process-type approach in Chinese, with reference to the classification criteria and description of SFL in Chinese in this study.

### Types of transitivity shifts for comparative analysis

Regarding the types of process shifts, drawing on the insights of Matthiessen's (2001, 2014) model on metafunctional variations, an important resource for doing transitivity analysis in translation studies, we examined process shifts from the following aspects:

a) Shifts among different process types;
b) Shifts within one process type;
c) The expansion and compression of process types:
  i. The expansion of process types:
    a. The addition of new process types of any kind;
    b. The extension of one process type into more than one kind;
  ii. The compression of process types:
    a. The omission of any process types;
    b. The compression of several process types into one.

The types of participant and circumstance shifts are studied where rank shifts occurred. Halliday divided a sentence into four categories from the bottom to top on the scale of rank: morpheme, word (Chinese character), word group (or phrase), and clause, to better analyze the lexicogrammar of one language. When one of these categories is transformed into the other at the rank level, there is a rank shift. Therefore, concerning participant and circumstance shifts, this research examined where there are obvious changes from the word group rank to the clause rank or *vice versa*.

The reason this study investigated such rank shifts first is that they tend to occur in English–Chinese translation when we study the transitivity shifts, for the two languages' different habits of expressing one thing bring out the inclination to use different rank scales. Therefore, when we compare the ST and TT and try to locate changes with the analytical unit of the transitivity system being the clause rank, rank shifts should be more visible than other types of participant and circumstance shifts. Moreover, as per Section “Types of transitivity shifts for comparative analysis”, each kind of process provides its own model for construing a particular domain of experience, with its own typically direct and indirect participants. This means that the shift from one process type to another must lead to the change of participants accordingly. Therefore, there is no point in simply analyzing such participant shifts caused by the shifts among different process types. Also, we should not pay too much attention to the shift among different types of circumstances, as they are not obvious enough, compared to those circumstance rank shifts.

### Register

Halliday (1985, p. 29) defined register as “a variety of language, corresponding to a variety of situations” and set three variables for describing and analyzing register: field (“what's going on in the situation”), tenor (“who is taking part in the situation”) and mode (“what role is being played by language and other semiotic systems in the situation”). These contextual factors determine the choice of linguistic forms (Halliday and Matthiessen, 2014, p. 34).

**Figure 2:**
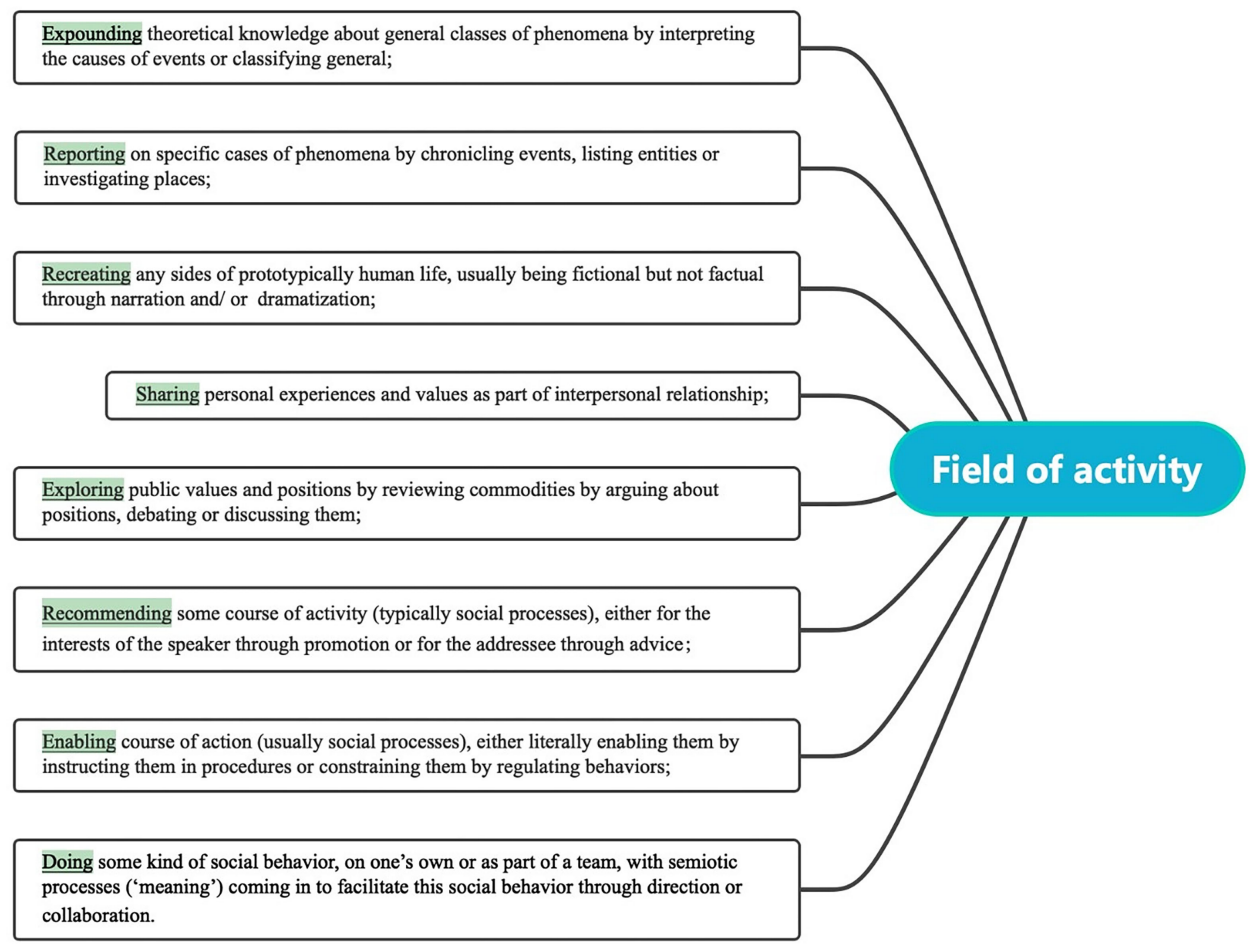
Definitions of eight fields of activity.

What this study considered in terms of the register of the factors motivating transitivity shifts is the field variable, more specifically, the fields of activity directly linked to the reproduction of experiential meaning. Matthiessen (2015, p. 55–56), developed eight main fields of activity (see Figure 2) to describe the nature of the activity that comprises the situation. As for the analytical unit for the fields of activity, one single sentence can serve as a self-contained unit. Therefore, ST and TT collected in Xi's books were analyzed in the unit of a sentence, being placed in the specific context of the sentence before the identification of fields, thus ensuring the effectiveness of the data.

## Methodology

This empirical research was conducted through a case study of the *Governance* volumes. First, the transitivity patterns of the ST and TT, and major tendencies of transitivity shifts were described through a comparative analysis, using a mixed approach of quantitative and qualitative analysis of the research materials. Thereafter, factors likely to motivate the tendencies were discussed with various translation examples.

We collected all the ACPP and their translations in the three *Governance* volumes as research materials, to address the research questions. The first volume (Xi, 2014a) explored President Xi's critical works, including the speeches, talks, interviews, instructions, and correspondence from November 15, 2012, to June 13, 2014. Volume two (Xi, 2017a) compiled Xi's critical speeches and writings from August 2014 to September 2017, and Volume three (Xi, 2020a) from October 2017 to January 2020. All the works were translated into English (Xi, 2014b, 2017b, 2020b) and released globally.

Our sample consists of a total of 318 items in ST and 305 translations, of which eight entries were quoted two times with the same translations, six were not translated, and one was rendered with two different translations. To avoid double-counting of transitivity shift types, the sample size should be 310 items with 305 translations. Concerning the analytical unit of clause for the transitivity system, there are 890 clauses, including the ranking clause (independent and subordinate clauses) and embedded clauses (functioning as participants despite their clause-like structure) in the ST and 824 clauses in the TT.

## Comparative analysis

This section presents a description of the overall transitivity patterns, followed by a detailed quantitative and qualitative analyses of the tendencies of transitivity shifts, and their impacts on the representation of experiential meaning, for providing answers to the first two research questions.

In terms of transitivity translation shifts and equivalence, Matthiessen (2001, p. 78–79), suggested that “equivalence is a matter of degree”, and “the degree to which two expressions in two different languages are equivalent will depend on how many features they share”. In this research, places with any type of shift as indicated in Section “Types of transitivity shifts for comparative analysis” were categorized as transitivity translation shifts, and otherwise as remaining transitively equivalent to the ST. The classification of transitivity shifts in this study covered processes, participants, and circumstances, all three parameters in the transitivity system which present the experiential meaning. Besides, for the nuclear parameter of transitivity of the process which plays the most important part in construing the experiential meaning, the three-level delicacy of process shifts was adopted in this research based on previous studies, which should illustrate as many features as the ST and TT can share in this regard.

As for the transitivity shift and equivalence tendency, compared to the transitivity patterns of the STs, the TTs are transitively shifted to a large degree. As shown in Table 1, 76 entries out of the total of 310 are equivalent, accounting for 24.52%, while nearly three-quarters of the translated poems or essays are transitively shifted from the original patterns, indicating that the experiential meaning was likely to be changed in the translations, consciously or unconsciously. However, the change of experiential meaning does not necessarily lead to poor quality and low accuracy of the translation. As Zhao (2006, p. 7), argued, “The syntactic differences sometimes make it difficult to get an ideal formal equivalence that carries the equivalent ideational meaning. In such cases, the translation shifts have to be considered to fit the translation well into the target language. A mechanical imitation of the original pattern can only lead to some unnatural translations”. Therefore, when the changes in experiential meaning were caused by the necessary transitivity translation shifts, there is no direct relationship between the transitivity shifts and the deterioration of translation accuracy or quality.

**Table 1:** Frequency and proportion of transitivity equivalence and shift in the three volumes.

**Equivalence and shift**	**Volume I**	**Volume II**	**Volume III**	**Total**	**Proportion**
Transitivity equivalence	32	27	17	76	24.52%
Transitivity shift	83	78	73	234	75.48%

### Tendency of process shifts

The process shifts we analyzed here are those among different process types, within one process type, and the expansion and compression of process types, as in Section “Types of transitivity shifts for comparative analysis”. Regarding the overall trend of how various process types construct the experiential meaning in ST and TT, Tables 2, 3 illustrate that among the six process types, the material clause is used the most, accounting for over 50% of the ST and TT. Relational process ranks the second and mental process the third, which proved again Halliday and Matthiessen (2004) statement that material, mental, and relational clauses constitute the three principal types that occur more often in many discourses. On the contrary, the verbal process is barely used both in the ST and TT.

**Table 2:** Frequency and proportion of process types in the ST.

**Process type**	**Material**	**Mental**	**Relational**	**Behavioral**	**Verbal**	**Existential**	**Total**
Volume I ST	175	52	71	6	2	7	313
Volume II ST	158	59	74	13	4	21	329
Volume III ST	141	22	63	12	3	7	248
Total ST	474	133	208	31	9	35	890
Proportion of each ST process type	53.26%	14.94%	23.37%	3.48%	1.01%	3.93%	

**Table 3:** Frequency and proportion of process types in the TT.

**Process type**	**Material**	**Mental**	**Relational**	**Behavioral**	**Verbal**	**Existential**	**Total**
Volume I TT	180	35	75	3	1	5	299
Volume II TT	169	34	75	11	1	14	304
Volume III TT	127	23	62	5	2	2	221
Total TT	476	92	212	19	4	21	824
Proportion of each TT process type	57.77%	11.17%	25.73%	2.31%	0.49%	2.55%	

**Figure 3:**
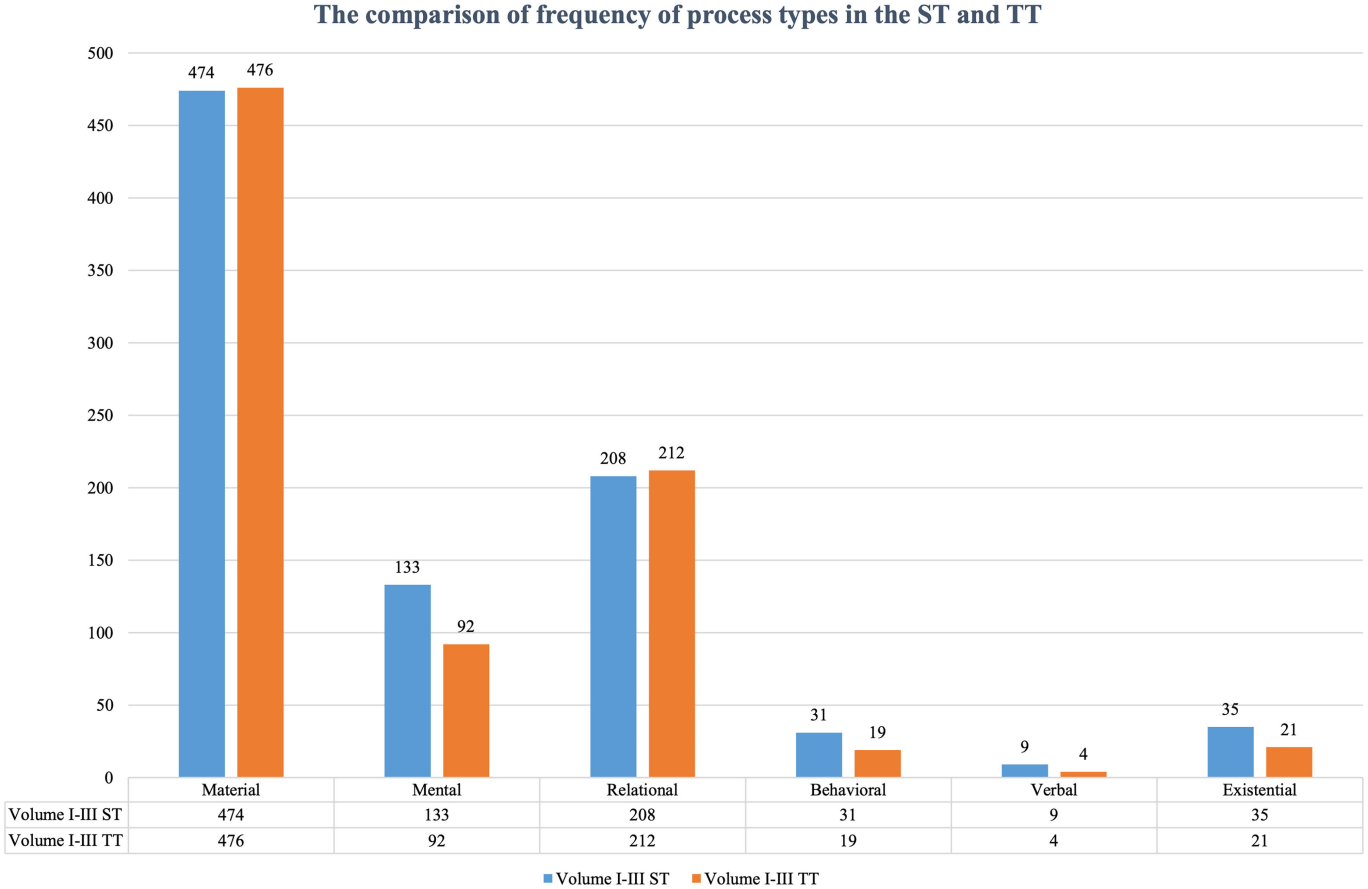
Comparison of frequency of process types in the ST and TT.

Concerning the distribution of process types in ST and TT, Tables 2, 3 reveal that material and relational processes are still exploited the most. If we compare the frequency of process types in the TT with the ST (see Figure 3), there are decreases in all the other four process types, except the material and relational ones. Typical political texts also characteristically use more material and relational clauses to construct meaning and build relationships among different entities. Therefore, literary texts and their TT in political texts can still not eliminate the texture of political texts themselves to a certain degree.

#### Tendency of shifts among different processes

Theoretically, there should be 30 categories of shifts among different processes, as listed in Table 4, but many types were not present in this study. Instead, the transformations from other process types to the three major types comprised a large proportion, with the change from other types to material process and relational process being the top two, accounting for over 46.25 and 32.5% respectively. Though the mental process is among the main process types, there were not too many processes being shifted into the mental one, making up less than 10%, much lower than the figure for material and relational processes. Besides, there were barely any verbal clauses in both the ST and TT, and, unsurprisingly, no process was transformed into the verbal type. Moreover, the table shows that the transformations among the three major types also comprised a large amount, with 37 material processes being shifted into relational ones, 35 relational processes into material ones, and 28 mental clauses into material types. Hence, the material process has strong tolerance and capacity for process shift among various types.

**Table 4:** Frequency of shifts among different processes.

**Types of process shift**	**Volume I**	**Volume II**	**Volume III**	**Total**	**Proportion**
The transformation from another type to MAP^a^	20	36	18	74	46.25%
MEP^b^ >^c^ MAP	8	13	7	28	
RP^d^ > MAP	11	17	7	35	
BP^e^ > MAP	0	1	0	1	
EP^f^ > MAP	1	5	4	10	
VP^g^ > MAP	0	0	0	0	
The transformation from another type to MEP	5	5	5	15	9.38%
MAP > MEP	4	4	3	11	
RP > MEP	1	0	1	2	
BP > MEP	0	0	0	0	
EP > MEP	0	1	0	1	
VP > MEP	0	0	1	1	
The transformation from another type to RP	20	24	8	52	32.50%
MAP > RP	15	15	7	37	
MEP > RP	4	5	1	10	
BP > RP	0	0	0	0	
EP > RP	1	3	0	4	
VP > RP	0	1	0	1	
The transformation from another type to BP	1	0	3	4	2.50%
MAP > BP	1	0	2	3	
MEP > BP	0	0	1	1	
RP > BP	0	0	0	0	
EP > BP	0	0	0	0	
VP > BP	0	0	0	0	
The transformation from another type to VP	0	0	1	1	0
MAP > VP	0	0	0	0	
MEP > VP	0	0	0	0	
RP > VP	0	0	0	0	
BP > VP	0	0	0	0	
EP > VP	0	0	1	1	
The transformation from another type to EP	0	5	1	6	3.75%
MAP > EP	0	4	1	5	
MEP > EP	0	1	0	1	
RP > EP	0	0	0	0	
BP > EP	0	0	0	0	
VP > EP	0	0	0	0	

^a^MAP, Material process.

^b^MEP, Mental process.

^c^“>” means the process type put before the symbol “>” is shifted into the other type after the symbol.

^d^RP, Relational process.

^e^BP, Behavioral process.

^f^EP, Existential process.

^g^VP, Verbal process.

The tendency of shifts from other process types to material and relational clauses suggests that the TT tends to reproduce the experience of the world to involve more changes in events and intensifies the relationships among different entities. The material clauses, as Halliday and Matthiessen (2004, p. 179) claimed, are “clauses of doing-&-happening: a ‘material' clause construes a quantum of change in the flow of events as taking place through some input of energy”, while relational processes are usually used to present qualities or features and to recognize identities. As material and relational processes are the types that other clauses tend to be shifted to, based on the nature of those two processes, we can infer that the TT tends to reproduce the experiential meaning to construe more flow of the changes of events and more relations among various objects.

Example 1:

ST: “很多科学研究要着眼长远, 不能急功近利, 欲速则不达” (Xi, 2017a, p. 276).

TT: “Haste makes waste. We will restrain from seeking immediate gains from research, but have our eyes on the future” (Xi, 2017b, p. 303).

Here is a case of process shifts from the material clause to the relational clause. The original Chinese poem means that if one pursues speed blindly, one cannot reach the destination. The second clause “则不达” is a material-transformative process type, one that shows “what is happening”, while that process is shifted into the relational process to identify the relationship between “欲速” (Haste) and “则不达” (waste). This relational-circumstantial-identifying process implies “欲速” (Haste) potentially leads to “则不达” (waste). By doing so, the translator tends to portray the potential bad consequence due to the Identified Participant of “haste”, thus changing the experiential meaning to a large degree. However, it does not necessarily lead to poor quality and accuracy of the translation, for here, the TT still fits the context properly.

Example 2:

ST: “不观时俗, 不察国本, 则其法立而民乱, 事剧而功寡” (Xi, 2017a, p. 117).

TT: “Where the customs of the times are ignored and fundamentals of the land in neglected, the people will fall into disorder, even when laws are made. And the ruler may be kept busy but will achieve little” (Xi, 2017b, p. 125).

In this case, there are process shifts from the relational clause to the material clause. The ancient Chinese essay is quoted to illustrate the importance of considering the realities and national conditions of China in choosing the path and system of the rule of law. In the ST, “事剧而功寡” means that if the ruler fails to base his work on national conditions, he will be busy, but the actions will remain fruitless. The original sentence consists of two relational-intensive-attributive clauses, characterizing the features of the two Carrier Participants “事” and “功” with the Attributes “剧” and “寡”. While the TT shifts these two relational processes to two material clauses to describe what was going on for the ruler, thus weakening the bonding relationship between the Carriers and their Attributes to a certain extent. However, the changes made by the translator are reasonable, as material clauses tend to be the major processes often used in a narrative.

#### Tendency of shifts within one process

For the tendency of shifts within one process, Table 5 demonstrates that TT shifts are more likely to occur within the material clauses than in any other type of process. Regarding the shifts within the material process, a much higher proportion of shifts comprises transformations among the subtypes of material-transformative clauses, where the Actor or Goal participant exists before the process is unfolded and these participants are transformed as the flow of events occurs in the process. Comparatively, only a few shifts are changes between material-transformative and material-creative clauses, where the Actor or Goal participant “is construed as being brought into existence as the process unfolds” (Halliday and Matthiessen, 2004, p. 184). Further, shifts barely take place within the three non-nuclear process types, similar to the shift pattern among different processes.

**Table 5:** Frequency and proportion of shifts within one process.

**Types of process shift**	**Volume I**	**Volume II**	**Volume III**	**Total**	**Proportion**
Shifts within MAP	17	17	17	51	68.00%
Creative < >^a^transformative	5	5	4		
Shifts within transformative subtype	12	12	12		
Shifts within creative subtype	0	0	1		
Shifts within MEP	2	2	4	8	10.67%
Like < >please	1	0	1		
Emotive < >perceptive	1	1	1		
Cognitive < >perceptive	0	1	2		
Shifts within RP	6	8	1	15	20.00%
Circumstantial < >possessive	1	0	0		
Intensive < >possessive	2	1	0		
Intensive < >circumstantial	2	3	1		
Shifts within intensive subtype: identifying < >attributive	1	4	0		
Shifts within BP	0	0	0	0	0%
Shifts within EP	0	0	0	0	0%
Shifts within VP	1	0	0	1	1.33%
Indicating>imperating	1	0	0		

^a^“ < >” means that two subtypes of one process type transform mutually.

The tendency of shifts being more among material-transformative clauses than between material-creative and -transformative clauses implies that the material domain of meaning in the ST does not change too much in translation. As Halliday and Matthiessen (2004, p. 184–185), explained about the difference between the two process subtypes, “in a creative clause the outcome is the coming into existence of the Actor (‘intransitive') or Goal (‘transitive')”. Therefore, when the two subtypes transform mutually, what has not emerged may be reproduced, as it has already existed, causing great changes in the material domain of meaning. However, the higher proportion of shifts among the single type of transformative clauses means that there are just changes in the ways of the outcome change, or in most cases, the changes between transitive and intransitive verbs as a result of the different language habits. For example, “死而后已”, a material-transformative-elaboration-intransitive clause, was translated as a material–transformative–extension–transitive process “till the heart beats it lasts”.

The tendency of a high proportion of shifts within the material and relational processes can influence the reproduction of experiential meaning. The change from one subtype to another within the same process type may bring about different configurations of various categories of participants, and different ways of interpreting experiential meaning.

Example:

ST: “穷则变, 变则通, 通则久。 面对我国经济发展新常态, 我们观念上要适应, 认识上要到位, 方法上要对路, 工作上要得力, 否则很难与时俱进抓好经济工作。” (Xi, 2017a, p. 233).

TT: “‘Limitations lead to change; changes lead to solutions; solutions lead to development.' We should be adaptive as we face the new normal. We should fully understand it, adopt the right measures, and make concrete efforts, so as to keep pace with the times in our economic work” (Xi, 2017b, p. 256).

“穷则变, 变则通, 通则久” was indicated by Present Xi to suggest that we should advance with the times and make changes accordingly to achieve sustainable economic development. Here, the two original clauses “变则通, 通则久” (changes can make things go smoothly and that will make things continue to develop and last forever) are two intensive-attributive-relational clauses, showing the features of the Carrier participant “变” and “通” with the Attribute participant “通” and “久”. In the TT, these two clauses were translated as two circumstantial-identifying-relational clauses: “changes lead to solutions; solutions lead to development”. Thus, the circumstantial phrase “lead to” builds a cause-and-effect relationship between the two Identified participants of “changes” and “solutions”, which is quite different from the characterizing function of an intensive-attributive clause.

Besides, as “lead to” can be followed only by a noun or nominal group, the adjective Attributes “通” and “久” are transformed as the nominal Identifier “solutions” and “development”, respectively, containing experiential meanings, which are rather different from the original texts. “通” refers to “通达的” (be easy to advance with no obstacles) and “久” means “长久的” (long-lasting). While here, the translator did not choose to stay too close to the original meanings of the two characters but reproduced them as “solutions” and “development”, implying good results in economic work and naturally connecting with what is said in the next sentence about the economic issue.

#### Tendency of the expansion and compression of process types

Table 6 indicates that the compression of process types comprises over 68% of the total shifts, much higher than the proportion of the expansion type. There are two kinds of compression of processes: the omission of process types, be it the deletion of original clauses and thus the missing of process types or the downgrading from the clause to the word group; and the compression of several process types into one, where the translator interprets the original meaning differently or there are language distinctions that force the translator to reproduce the ST otherwise. Much of the compressions occur when the repetition of certain verbs or verb-clauses in the ST is simplified or even omitted in the TT. Besides, there is the tendency of shifts from a higher linguistic rank to a lower one, in particular, the nominalization and the process types being transformed into a participant or circumstance parameter, which will be discussed in Section “Tendency of participant and circumstance nominalization”.

**Table 6:** Frequency and proportion of the expansion and compression of process types.

**Types of process shift**	**Volume I**	**Volume II**	**Volume III**	**Total**	**Proportion**
Expansion of process types	26	19	13	58	31.18%
Compression of process types	44	50	34	128	68.82%

The tendency of the compression of process types can change the intensiveness of meaning realized in clauses. As Chinese relies much on verbs and verbal groups to construe the experience of the world, clauses of different kinds tend to occur in which more information is embedded, causing the high intensity of meaning expressed in one sentence. Moreover, the Chinese language prefers repetition [For instance, “知之者不如好之者, 好之者不如乐之者” (Xi, 2014a, p. 406) and “不知人之短, 不知人之长, 不知人长中之短, 不知人短中之长, 则不可以用人, 不可以教人” (Xi, 2014a, p. 418)] and the Chinese people regard it as a rhetoric device to emphasize what they would like to convey and to intensify their feelings or the power of language. However, in English, repetition and similar expressions being used altogether simultaneously in most cases are not the preferable options and tend to be omitted or shifted in one sentence.

Here is a case of compressing types of process, but it involves only a shift within a single transitivity process, the relational one.

ST: “第一, 深学笃用, 通过示范引领让干部群众感受到新发展理念的真理力量。 知之愈明, 则行之愈笃。” (Xi, 2017a, p. 219).

TT: “First, officials should set an example by conscientiously studying the new development concepts and making earnest efforts to apply them, to show the strength of these new concepts to other officials and the general public. ‘A clearer understanding of new concepts makes actions stronger and more targeted”' (Xi, 2017b, p. 241).

In this example, the ST (literarily means the clearer the understanding, the more solid the practical action) is cited in a context where President Xi calls for officials at all levels to make efforts to learn five new development concepts to enable them to take root and become a common practice. The TT reduces the two relational clauses into one, linking them with a new relational process, thus stabilizing and highlighting the relationship between the two participants of “a clearer understanding of new concepts” and “actions” with the attributive “stronger and more targeted” as an attribute of “actions”. While the first relational clause in the ST is nominalized into a phrase group, condensing information within the shift from a clause to a nominal group.

### Tendency of participant and circumstance nominalization

Nominalization refers to the downgrading of the rank from verbs (verbal groups) that serves the six processes to nouns (nominal groups). In this section, we will discuss the nominalization of verbs and verbal phrases serving all process types, which function as participants and circumstances in a clause after the change.

**Figure 4:**
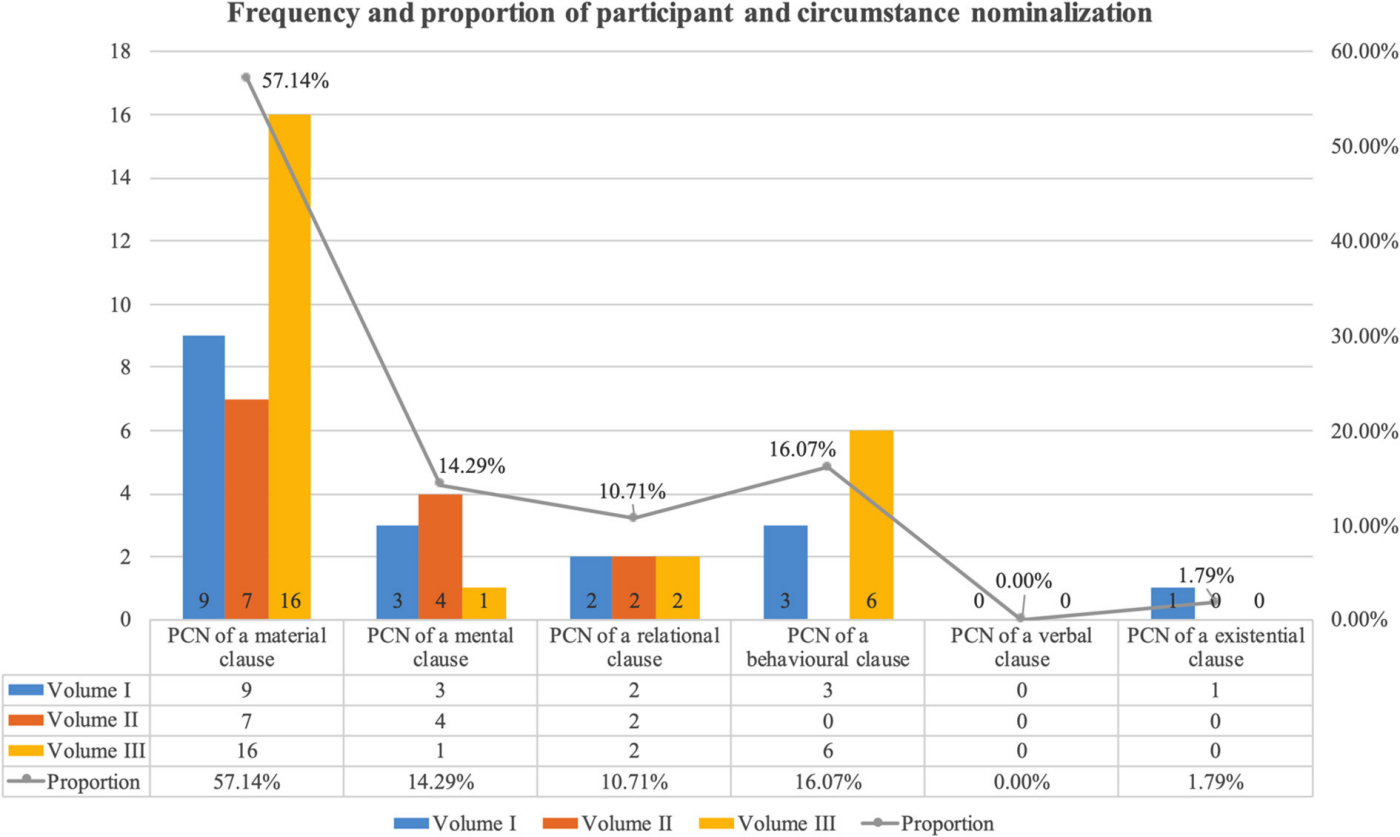
Frequency and proportion of participant and circumstance nominalization in the ACPP^1^.

Regarding the distribution and frequency of nominalizations, first, we can see from Table 7 and Figure 4 that there are 39 participant nominalizations, 17 circumstance nominalizations, and nine nominalizations of other types. Participant nominalizations are slightly higher than circumstance nominalizations. Besides, there is the tendency for participant and circumstance nominalizations to occur mainly where there are the core process types in the ST, accounting for over 87%. Among them, the material clause is the easiest that is shifted to the nominal group compared to the other types of process, amounting to 60.71%, followed by relational, mental, and behavioral clauses.

**Table 7:** Frequency of nominalization in the three volumes of *Governance*.

**Types of nominalization**	**Volume I**	**Volume II**	**Volume III**	**Total**
Participant nominalization	10	7	22	39
Participant nominalization of a material clause	5	1	15	
Participant nominalization of a mental clause	3	4	2	
Participant nominalization of a relational clause	1	2	2	
Participant nominalization of a behavioral clause	0	0	3	
Participant nominalization of a verbal clause	0	0	0	
Participant nominalization of an existential clause	1	0	0	
Circumstance nominalization	8	6	3	17
Circumstance nominalization of a material clause	4	6	3	
Circumstance nominalization of a mental clause	0	0	0	
Circumstance nominalization of a relational clause	1	0	0	
Circumstance nominalization of a behavioral clause	3	0	0	
Circumstance nominalization of a verbal clause	0	0	0	
Circumstance nominalization of an existential clause	0	0	0	
Others	6	1	2	9
Nominalization of a material clause	4	0	2	
Nominalization of a mental clause	1	0	0	
Nominalization of a relational clause	1	1	0	
Nominalization of a behavioral clause	0	0	0	
Nominalization of a verbal clause	0	0	0	
Nominalization of an existential clause	0	0	0	
Total frequency	24	14	27	65

For instance, the original text “物必先腐, 而后虫生” (Things must rot first, and then insects would grow) (Xi, 2014a, p. 16) consists of two material clauses. While in the translation “Worms can only grow in something rotten” (Xi, 2014b, p. 17), one of the material clauses was rendered as “something rotten”, functioning as a circumstance in the whole clause following the proposition “in”. For the participant nominalization of a material clause, one typical example is that while rendering “宽一分, 民受赐不止一分” (showing more mercy to the people and they will be benefited a lot) (Xi, 2017a, p. 148), the translator shifted the original material clause “宽一分” to a nominal group in the TT as “A bit of leniency on my side” (the whole translation is “A bit of leniency on my side will benefit the people”) (Xi, 2017b, p. 162), serving as the Actor participant in the new material clause.

Proceeding to the nominalization of the three subsidiary processes, no verbal clauses are shifted into the nominal groups in the three volumes. Participant and circumstance nominalization of a behavioral clause comprises a greater proportion than that of the other two subsidiary process types, accounting for over 10%. However, all the cases come from the three sentences of “耳闻之不如目见之, 目见之不如足践之” (Xi, 2014a, p. 417), “耳得之而为声, 目遇之而成色” (Xi, 2020a, p. 507) and “听言观行” (Xi, 2020a, p. 124). The five verbal groups (“耳闻”, “目见”, “耳得”, “目遇”, and “听言”) serving the behavioral embedded clauses were rendered as nominal participants: “hearing”, “seeing” (Xi, 2014b, p. 467), “ear”, “eye”, and “words” (Xi, 2020b, p. 587). With such scattered examples, it might not be typical and representative enough to say that participant and circumstance nominalizations of a behavioral clause tend to occur more often than other subsidiary processes.

Further, a few cases are eventually shifted into nominal phrases, not being a clause with participants and circumstances anymore, thus falling under the other types of nominalization. For example, “四维不张, 国乃灭亡” (Xi, 2014a, p. 168) was translated as “Propriety, righteousness, honesty and a sense of shame—the four anchors of our moral foundation, and a question of life and death for the country” (Xi, 2014b, p. 188). The whole translated sentence consists of several nominal phrases, downgrading the rank of a clause to the word group. However, as the translation itself is not a clause, there are of course no participant and circumstance element components in it.

The tendency of participant and circumstance nominalization of the three core process types, in particular the material clause, can change the density of experiential meaning expressed in one clause. The ST tends to use more verbal groups or verbs that constitute process types in a sentence to construe experiential meaning in Chinese, while the English translations tend to transform parts of those processes into nominal groups or nouns serving as participants and circumstances, leaving fewer processes in a clause. The fewer the processes with more participant or circumstance elements in a clause, however, the denser the experiential meaning realized in one clause and the more highlighted the meaning of the processes left after the shifts.

Example 1. Participant nominalization:

ST: “尚贤者, 政之本也。” 各级党委和政府要认真贯彻党和国家关于留学人员工作的方针政策, 更大规模、更有成效地培养我国改革开放和社会主义现代化建设急需的各级各类人才。 (Xi, 2014a, p. 61).

TT: “‘Exaltation of the virtuous is fundamental to governance.' Party committees and governments at all levels must earnestly implement Party and government policies concerning students and scholars studying abroad, and train more effectively and on a larger scale all kinds of talented people badly needed by our reform, opening up and modernization” (Xi, 2014b, p. 67).

In this case, “者” is a modal particle that does not contain real meanings. “尚贤者” is an embedded identifying-relational clause that functions as a participant in the whole sentence “尚贤者, 政之本也” (respecting the virtuous is the root of governance). In the English translation, “尚贤者” was reproduced as a nominal group “exaltation of the virtuous”, instead of rendering it as an embedded clause with a verbal group to be consistent with the original structure. The experiential meaning realized in two clauses in the ST now is condensed into one clause in English, stressing the most important process based on the translators' understanding.

Example 2. Circumstance nominalization:

ST: “志不立, 天下无可成之事。理想信念动摇是最危险的动摇, 理想信念滑坡是最危险的滑坡。” (Xi, 2017a, p. 34).

TT: “‘Without resolve, one can accomplish nothing.' The wavering of one's faith and ideal is the most dangerous risk” (Xi, 2017b, p. 34).

In this example “志不立, 天下无可成之事” (if ambition is not established, nothing can be done in the world), one of the two material clauses “志不立” was reproduced as a circumstance element “without resolve” in translation. So, there is only one material process left, compressing the density of meaning in that single clause.

## Discussion on factors motivating the transitivity shifts

In this section, possible factors motivating process, participant and circumstance shifts will be discussed, including the consideration of specific contextual elements closely related to the choice of the transitivity system, namely, the register variable of field.

### Factors motivating process shifts

#### Factors to shifts among different process types

The tendency of the shifts from other processes to material and relational process types is closely related to the genre of the text. When literary texts are translated into political texts, they inevitably catch certain features of political discourse where ^1^ material and relational processes tend to be exploited more frequently than mental, verbal, behavioral, and existential processes in novels, poems, and other literature, to construct the experience of the events and build relationships among different entities. The ACPP in most cases is quoted in ST and TT to justify, illustrate or emphasize the author's political viewpoints and his philosophy of governance. These citations constitute only a small part of the whole political work and some are only a part of a complex sentence; therefore, they often inevitably bear the transitivity characteristics of political texts, more or less.

#### Factors to shifts within one process type

First, the tendency of the large proportion of shifts within the material clause is connected to the levels of delicacy. According to Halliday and Matthiessen (2004, p. 169–248), eight subtypes of material processes are used often (listed below), making three levels of delicacy.

1. Creative-intransitive material clause.
2. Creative-transitive material clause.
3. Transformative-elaboration-intransitive material clause.
4. Transformative-elaboration-transitive material clause.
5. Transformative-extension-intransitive material clause.
6. Transformative-extension-transitive material clause.
7. Transformative-enhancement-intransitive material clause.
8. Transformative-enhancement-transitive material clause.

Halliday also categorized other minor subtypes and set four delicacies to classify the material process more comprehensively. Such delicacies neutrally bring about more subtypes for material clauses compared to other processes; hence, more shifts within this process type are likely, especially in the political discourse where material processes comprise a large proportion. This is also partly why shifts within the relational process also account for a greater proportion, compared to the shifts within the other four types of processes. Secondly, material processes constitute more than 50% in both ST and TT and the large number of material clauses provide room for shifts within this process type itself.

#### Factors behind the expansion and compression of process types

There is a tendency toward a large proportion of the process compression resulting from the differences between Chinese and English. The former is a language of parataxis that prefers construing the experience of the world with various classes of verbs and verbal groups–hence the creation of more clauses of different kinds, in particular the embedded clauses that serve as participant and circumstance elements for their ranking clauses. By contrast, English is a language of hypotaxis that values the sequence of different levels of ranks and does not prefer the use of too many verbs or verbal groups in one clause, leading to many Chinese processes being cut in English clauses. Further, when the rhetorical device of repetition is used in Chinese to stress the original meaning or to show the power of the language itself, processes are also inclined to be reduced or omitted, for that figure of speech is sometimes regarded as redundancy in English expressions.

### Factors motivating participant and circumstance shifts

First, the tendency of participant and circumstance nominalization is ascribed to the different language habits. English prioritizes the use of nouns and nominal groups to present an experience of the world, while in Chinese verbs and verbal phrases are always the first choices. This different tradition of configuration language structure results in the inclination of replacing Chinese processes with nominal phrases. Second, the difference in logical structure between the two languages can also result in the rank shift of nominalization. English prefers to present things systematically in one sentence, while Chinese tends to describe them linearly. That therefore often makes Chinese clauses turn into one English sentence with several embedded clauses. Nominalization is one of the ways to realize such a trend, changing the Chinese verbal groups that serve processes in clauses to English nominal groups that can form embedded clauses in one complicated sentence. Third, the high frequency of material and relational processes distributed in the ST can also be the reason for participant and circumstance nominalization in many cases occurring in these two types of clauses in the ST.

### Field factors motivating transitivity shifts

**Figure 5:**
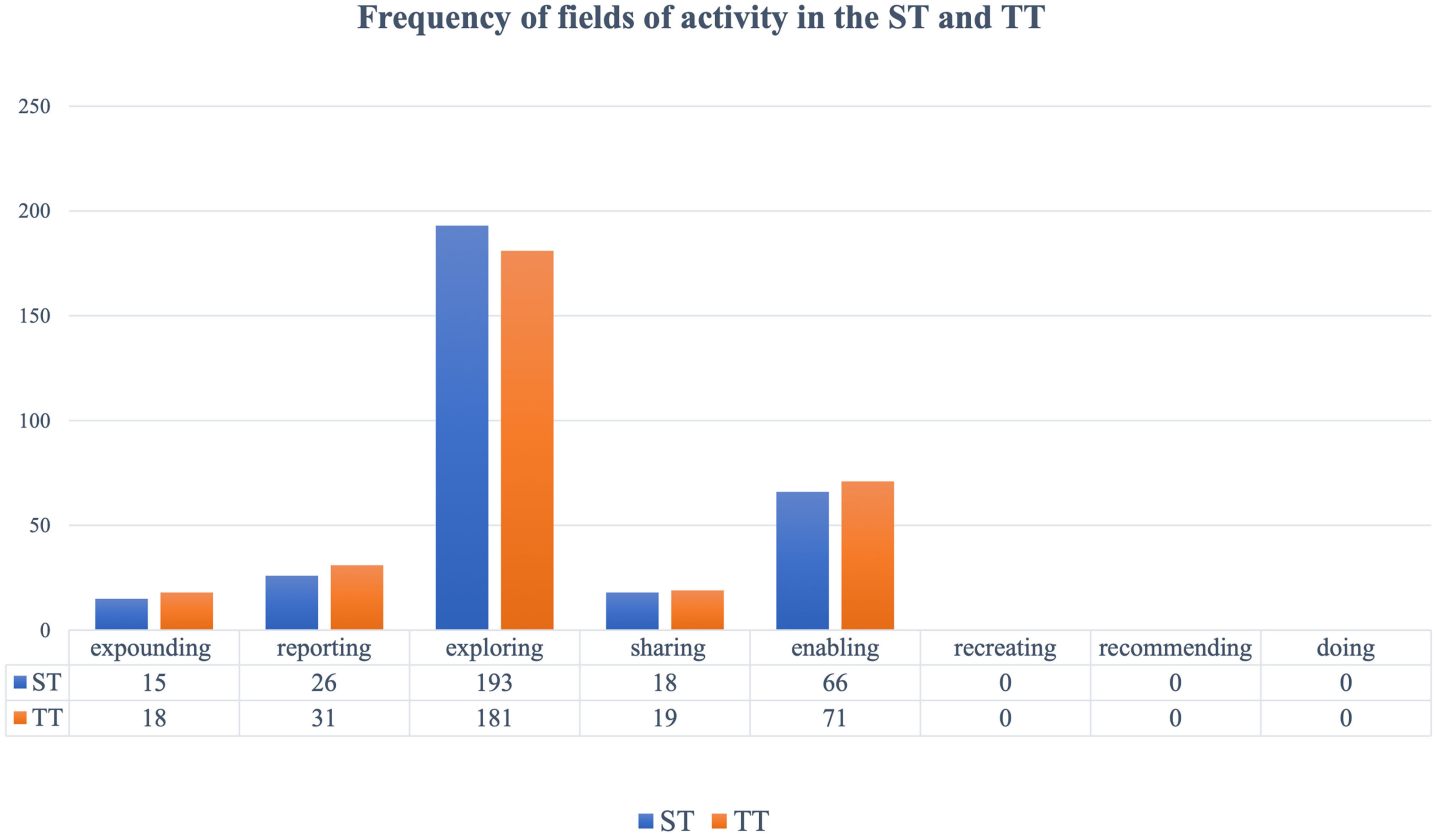
Frequency of fields of activity in the ST and TT.

The study found the existence of only five fields of expounding, reporting, exploring, sharing, and enabling in the materials (see Table 8 and Figure 5). Among them, exploring and enabling fields are used more often, comprising about 80% both in ST and TT, because in most cases, as shown in the following instances, the ACPP was quoted by President Xi to argue, discuss, or explore certain values and opinions publicly, to offer instructions or to warn people to avoid doing unwise things.

**Table 8:** Frequency and proportion of fields of activity.

**Fields of activity**	**ST**	**Proportion of each field in ST**	**TT**	**Proportion of each field in TT**
Expounding	15	4.72%	18	5.63%
Reporting	26	8.18%	31	9.69%
Exploring	193	60.69%	181	56.56%
Sharing	18	5.66%	19	5.94%
Enabling	66	20.75%	71	22.19%
Recreating	0	0	0	0
Recommending	0	0	0	0
Doing	0	0	0	0
Total	318	100%	320	100%

Example 1. Field of exploring:

ST: “一花独放不是春, 百花齐放春满园。 如果世界上只有一种花朵, 就算这种花朵再美, 那也是单调的。” (Xi, 2014a, p. 258).

TT: “As we often say in China, a single flower does not make spring, while one hundred flowers in full blossom bring spring to the garden. All countries are closely linked and share converging interests. They should both pool and share their strength” (Xi, 2014b, p. 283).

This example of exploring the field is part of Xi's address at the headquarters of UNESCO in 2014. Here, the prose in ST was invoked to stress the idea that all civilizations and cultures together make the world diverse and beautiful and thus, all countries need to work together. That functional nature of activity remains unchanged in TT–hence the same field of exploring in TT.

Example 2. Field of enabling:

ST: “…………非公有制经济要健康发展, 前提是非公有制经济人士要健康成长。……我们共产党内对领导干部也是这样要求的, 而且要求得更严……我们都要 自强不息, 止于至善。…………” (Xi, 2017a, p. 263).

TT: “…the healthy development of the private sector depends on those working in the sector…In the CPC, officials are required to do likewise, and the bar is even higher…We should constantly improve ourselves and strive for excellence” (Xi, 2017b, p. 287).

This sentence about enabling field comes from Xi's speech during the 4^th^ Session of the 12^th^ CPPCC National Committee in 2016. The ancient Chinese prose here was quoted to instruct that Party members needed to keep developing themselves, which in turn will lead to the improvement of the people and a robust economy. The functional nature of saying these words in both ST and TT is to provide instructions and form the field of enabling.

Statistics show that changes in the field of activity can lead to process shifts. Although Table 9 suggests that only about 14% of the fields of activity in ST were changed in their translations, this study found that where there is a shift in the field of activity, it is a process shift. In other words, when the original contextual field of activity is transformed in the TT, the process also tends to be changed to play different functions accordingly.

**Table 9:** Frequency and proportion of fields of activity equivalence and shift.

**Equivalence and shift**	**Volume I**	**Volume II**	**Volume III**	**Total**	**Proportion**
Fields of activity equivalence	97	101	76	274	85.63%
Fields of activity shift	19	11	16	46	14.38%

Example 1:

ST: “用人得当, 首先要知人。知人不深、识人不准, 往往会出现用人不 当、用人失误。 不知人之短, 不知人 之长, 不知人长中之短, 不知人短中之长, 则不可以用人, 不可以教人。” (Xi, 2014a, p. 418).

TT: “To employ officials, the most important thing is to know them. If we do not know them thoroughly and accurately enough, we may employ them inappropriately ‘Having no idea of a person's weakness and strength, the weak part of the strength or the strong part of the weakness, we have no ground for appointing or even training that person”' (Xi, 2014b, p. 469).

Here, the ACPP quoted in the ST is expressed in the field of enabling one to regulate one's behavior and avoid people employing talents wrongly. The imperative sentence with the imperative mood of “不可以” clearly tells the readers what the people cannot do, while the other clauses of “不知人之短, 不知人之长, 不知人长中之短, 不知人短中之长” list the prerequisites. While in the TT, the field of enabling was translated as the field of reporting, presenting the description that it is groundless to employ someone we do not know. While the two material clauses “不可以用人, 不可以教人” was rendered as a relational process “we have no ground for appointing or even training that person”, there emerges the transformation from the enabling field to the reporting field, so that the whole sentence sounds less peremptory. Such process shift is brought into existence in the interaction between the text and context– in this case, the change of process and field of activity.

Example 2.

ST: “功崇惟志, 业广惟勤。我国仍处于并将长期处于社会主义初级阶段, 实现中国梦, 创造全体人民更加美好的生活, 任重而道远……” (Xi, 2014a, p. 49).

TT: “‘One must both have great ambition and make tireless efforts to achieve great exploits.' China is still in the primary stage of socialism and will remain so for a long time to come. There is still much to do and a long way to go before we can realize the Chinese Dream and create a better life for all our people...” (Xi, 2014b, p. 60).

Similarly, in this example, the expounding field of “功崇惟志, 业广惟勤” was translated as the enabling field by using the modal verb “must”. Here, “惟” refers to “lie in” and the original quote contains two circumstantial-attributive-relational clauses, literally meaning fruitful achievements lie in ambition and great business lies in diligence. However, they were translated into one possessive-attributive-relational clause and two material clauses in English. The shift within the relational process type here is partly caused by and also leads to the change in the field of activity.

## Conclusions

### Major findings

Through a case study of Xi's *Governance* volumes and comparing the transitivity systems of ST and TT, this research found that in general, there exists a high proportion of various process shifts as well as participant and circumstance rank shifts, which can lead to a certain degree of experiential meaning shift in the translations, even if we ignore the necessary shifts caused by language differences. Specifically, several transitivity shift tendencies were discovered, as follows:

First, there are three major tendencies of process shifts.

a) The tendency of shifts among different processes is that other process types tend to be changed to material and relational clauses. Such a trend suggests that TT tends to reproduce the experiential meaning such that it involves more representations of the flow of events and the creation of relationships among entities. In turn, the much lower proportion of shifts from other clauses to the mental one, compared to that of the other two principal processes, demonstrates that personal perception, emotion, consciousness, or desire are not prone to be reproduced. Instead, it is the experience of the physical realm that the TT is likely to focus on in translating experiential meaning. Besides, the process type distribution of ACPP translations in the political texts is very similar to that of the ST, with material and relational clauses being the two most used processes. As typical political texts too employ more material and relational processes to construe experiences, it may be reasonable to say the translation of literary texts in political texts still bears the texture of political discourse itself to a certain degree.
b) The tendency of shifts within one process is that there is a large proportion of shifts occurring within material clauses. Among them, more shifts can be seen among material-transformative clauses, which can lead to configurational changes in various categories of participants and different ways of interpreting experiential meaning. However, the tendency of more shifts among material-transformative clauses than between material-creative and -transformative clauses suggests that the material domain of meaning in the ST does not change too heavily in translation, for, in most cases, those are the shifts between transitive and intransitive verbs because of the different language habits.

This study also found that compression, rather than expansion of processes, is the tendency in translating the ACPP in political texts, including the omission of processes and the compression of several process types into one. It indicates the TT is inclined to simplify certain experiences to make the language acceptable in English, by omitting unnecessary processes or condensing several processes into one. Such a tendency can bring about a change in the intensiveness of experiential meaning realized in clauses.

Second, there is the tendency for participant and circumstance rank shifts of nominalization. It means that verbs or verbal groups in a clause are changed to nouns or nominal groups that function as participants or circumstances. This trend can change the density of experiential meaning conveyed in one clause. The fewer processes with more participants or circumstances in a clause, the denser the experiential meaning realized in one clause and the more emphasis on the meaning expressed in processes.

Regarding possible factors motivating the transitivity shifts, this study discovered that the genre of political texts, language differences, and the distribution of process types in ST and TT as well as the delicacy level of the transitivity system can lead to the aforesaid transitivity shift tendencies. Besides, we can also go beyond to consider the motivations for those shifts from the contextual perspective of the field in the register theory. Regarding the field factors to transitivity shifts, it can be seen from the statistics where there was a change of the field of activity, there was a process shift in translation because when the field is shifted, the process also tends to be transformed to play different functions accordingly.

### Research limitations

These findings of the study have to be seen in the light of some limitations. The first is the inability to cover the fourth volume of *Governance* and its English translation released in July 2022. Due to time constraints, we could study the ACPP in the first three volumes of the paper alone, leaving space for future systematic functional translation studies to explore more materials. Secondly, the identification of transitivity process types in this research is based on empirical studies and is therefore subject to a certain bias, which may potentially influence the tendencies of transitivity shifts. However, whenever there is ambiguity in determining the process type for one clause, we would put the verb or verbal group into a border unit of the sentence or even the whole passage, rather than think only of the clause itself. Halliday (2002b) suggested in this regard the use of “trinocular perspective” which involves the combination of semantics, lexicogrammar, as well as phonology and graphology. Besides, it is proven by previous research (e.g., Halliday, 2002a; Halliday and Webster, 2004) that extralinguistic factors, such as register, are also directly related to the pattern of process types. Therefore, we also considered the contextual elements, if necessary, in choosing the most suitable process type for the clause. The third is the lack of previous studies on the topic of ACPP translations in *Governance* or other political texts with transitivity. Although the transitivity system has been widely used in literary translation studies, prior research on the translation of ACPP in Xi's works or other political texts is limited. However, this research proved that transitivity could offer a perspective on such a topic for experiential meaning, as the primary mode of metafunctional meaning is inherent in all languages, regardless of the differences in text genre. More theories are expected on this topic through further research. Another limitation lies in the scope and depth of the analysis and discussions. This study focuses on transitivity shifts within the experiential metafunction. However, there may be room for shifts between experiential and other metafunctions, leaving space for other systematic functional translation studies to explore. Moreover, as Li (2005, p. 98) suggested long before, the linguistic patterns of translation shifts will only serve for a starting point for investigating the reasons behind such shifts. To penetrate deeply into this area, the ultimate goal of translation shift studies will be the exploration of translation norms motivating shift tendencies, thus the translators' and even patrons' overall perception to literary translation in political texts during a certain period. Therefore, more empirical studies are expected for further advancement in this research field.

## Data availability statement

The original contributions presented in the study are included in the article/Supplementary material, further inquiries can be directed to the corresponding author/s.

## Author contributions

QL contributed to the conception of the work, data collection, analysis and interpretation, drafted the article, and made revisions. XL helped supervise the project and acquired financial support for the project. All authors discussed the results and contributed to the final manuscript.

## Funding

This work was supported by the Fundamental Research Funds for the Central Universities: B210202172. The funders had no role in study design, data collection and analysis, decision to publish, or preparation of the manuscript.

## Conflict of interest

The authors declare that the research was conducted in the absence of any commercial or financial relationships that could be construed as a potential conflict of interest.

## Publisher's note

All claims expressed in this article are solely those of the authors and do not necessarily represent those of their affiliated organizations, or those of the publisher, the editors and the reviewers. Any product that may be evaluated in this article, or claim that may be made by its manufacturer, is not guaranteed or endorsed by the publisher.

## Footnotes

^1^“PCN” in Figure 4 stands for participant and circumstance nominalization.
