# Supplementary material for: Reproducing experiential meaning in translation: A systemic functional linguistics analysis on translating ancient Chinese poetry and prose in political texts
Source: Front Psychol. 2022 Nov 24;13:1029187. doi: 10.3389/fpsyg.2022.1029187 (PMC9731279; doi:10.3389/fpsyg.2022.1029187)
Supplement: Supplementary file 1 [file Table_1.docx]

## Supplementary table

Supplementary Table 1. English transitivity process types

| **Process type** | **Meaning** | **Subtypes** | **Examples of verbs** |
| --- | --- | --- | --- |
| Material process | clauses of doing-and-happening; | Creative-intransitive material clause | appear, emerge; occur, happen, take place |
|  |  | Creative-transitive material clause | create, make, prepare, assemble, build, write, cook, found, establish, open, set up; |
|  |  | Transformative-elaboration-intransitive material clause | erupt, cut, glimmer, roar; increase, reduce, compress, enlarge, extend, form, shape |
|  |  | Transformative-elaboration-transitive material clause | destroy, remove, rule, govern; cut, compress, enlarge, extend, expand; form, shape |
|  |  | Transformative-extension-intransitive material clause | Give, offer, advance, leave, deny, serve, supply, provide, present, acquire |
|  |  | Transformative-extension-transitive material clause | join, meet; assemble, accumulate, collect, separate; spread |
|  |  | Transformative-enhancement-intransitive material clause | come, go; approach, arrive, reach, return; depart, leave; walk, jump, fly; |
|  |  | Transformative-enhancement-transitive material clause | bring, take, fall/fell, rise/raise (motion: place); walk, jump, fly; |
| Mental process | clauses of sensing; | Perceptive-like mental clause; | perceive, sense; see, notice, glimpse; hear, overhear; feel; taste; smell |
|  |  | Perceptive-please mental clause; | (assail) |
|  |  | Cognitive-like mental clause; | think, believe, expect, consider, know; understand, realize, appreciate; imagine; guess; doubt; forget; |
|  |  | Cognitive-please mental clause; | strike, occur to, convince; remind, escape; puzzle, intrigue, surprise |
|  |  | Desiderative-like mental clause; | want, wish, would like, desire; hope (for); plan; decide, resolve, determine; agree, refuse |
|  |  | Desiderative-please mental clause; | (tempt) |
|  |  | Emotive-like mental clause; | like, fancy, love, adore, dislike, hate, despise; rejoice, exult, grieve, mourn, regret; fear; enjoy |
|  |  | Emotive-please mental clause; | attract, please, disgust, offend; delight, depress; alarm, scare, shock, comfort, encourage; amuse, interest, fascinate, bore, worry |
| Relational process | clauses of being; | intensive-attributive relational process | be (eg. Shakespeare is a writer), feel, become, remain, prove, confirm, weigh, cost, measure |
|  |  | intensive-identifying relational process | be (eg. Shakespeare is the writer), play, act as, function as, serve as, express, feature, comprise, feature, include |
|  |  | possessive-attributive relational process | be, have (eg. Mike has a cap. ), belong |
|  |  | possessive-identifying relational process | be (eg. The cap is Mike’s.), own |
|  |  | circumstantial-attributive relational process | be (eg. The meeting will be on Monday.), depend on, concern, is concerned with, deal with, become (for comparison) |
|  |  | circumstantial-identifying relational process | be (eg. The time of the meeting will be on Monday.), resemble, match, ﬁt; cover, touch upon, take up, discuss, support |
| Behavioural process | clauses of behaving; |  | look, watch, stare, listen, think, worry, dream, cry, laugh, smile, frown, sigh, breathe, sleep, sing, dance, |
| Verbal process | clauses of saying; |  | praise, slander, blame, criticize, say, tell, convince (that), persuade (sb that), promise (that), ask, order |
| Existential process | clauses of existing; |  | exist, remain, follow, ensue, sit, stand, lie; happen, take place; prevail |
